# Psychology in Tuberculosis

**Published:** 1920-09-04

**Authors:** 


### Psychology in Tuberculosis.

Here are a few notable quotations from the recently published book on "Industrial Colonies and Village Settlements for the Consumptive," by Sir German Woodhead and Dr. P. C. Varrier-Jones:—

"A consumptive must learn to be a consumptive; that is the hard lesson he has to learn. But those who have to deal with consumptives have also much to learn. It is only by the most careful study of the patient's outlook, by assiduous care in placing him in surroundings where he may work without worry, where the trials of everyday life may be reduced, that the irritability of the consumptive's mind can be assuaged."

The authors offer an interesting speculation as to the connection of the disease with the mental condition of the Archduke Francis Ferdinand. He had a typically phthisical temperament—he was unbalanced in everything, he bore a grudge against numberless people, and "an unparalleled contempt for the world" had become a characteristic feature of his whole nature. "Who can say," they ask, "whether the toxin of the tubercle bacillus, influencing this man's relations with his fellows, may not have precipitated the European upheaval?"

Discussing the disease in its relation to the course of civilisation, the authors remark: "It has directed or checked the migration of races, it has preserved the temperate regions for the white man, with his greater powers of resistance, it has seemed in many instances to have been the handmaid of genius, it has precipitated great catastrophes."

